# Potential pitfalls in MitoChip detected tumor-specific somatic mutations: a call for caution when interpreting patient data

**DOI:** 10.1186/1471-2407-10-597

**Published:** 2010-10-30

**Authors:** Malliya Gounder Palanichamy, Ya-Ping Zhang

**Affiliations:** 1 Laboratory for Conservation and Utilization of Bio-resources, Yunnan University, 2 North Green Lake Street, Kunming 650091, China; 2 State Key Laboratory of Genetic resources and Evolution, Kunming Institute of Zoology, Chinese Academy of Sciences, Kunming 650223, China

## Abstract

**Background:**

Several investigators have employed high throughput mitochondrial sequencing array (MitoChip) in clinical studies to search mtDNA for markers linked to cancers. In consequence, a host of somatic mtDNA mutations have been identified as linked to different types of cancers. However, closer examination of these data show that there are a number of potential pitfalls in the detection tumor-specific somatic mutations in clinical case studies, thus urging caution in the interpretation of mtDNA data to the patients. This study examined mitochondrial sequence variants demonstrated in cancer patients, and assessed the reliability of using detected patterns of polymorphisms in the early diagnosis of cancer.

**Methods:**

Published entire mitochondrial genomes from head and neck, adenoid cystic carcinoma, sessile serrated adenoma, and lung primary tumor from clinical patients were examined in a phylogenetic context and compared with known, naturally occurring mutations which characterize different populations.

**Results:**

The phylogenetic linkage analysis of whole arrays of mtDNA mutations from patient cancerous and non-cancerous tissue confirmed that artificial recombination events occurred in studies of head and neck, adenoid cystic carcinoma, sessile serrated adenoma, and lung primary tumor. Our phylogenetic analysis of these tumor and control leukocyte mtDNA haplotype sequences shows clear cut evidence of mixed ancestries found in single individuals.

**Conclusions:**

Our study makes two prescriptions: both in the clinical situation and in research 1. more care should be taken in maintaining sample identity and 2. analysis should always be undertaken with respect to all the data available and within an evolutionary framework to eliminate artifacts and mix-ups.

## Background

Mitochondrial DNA technology plays an exciting role in medical research especially the high throughput MitoChip for mtDNA mutation detection in cancer. In the past few years the MitoChip technique has uncovered a large number of mtDNA mutations in human head and neck, adenoid cystic carcinoma, sessile serrated adenoma, and lung primary tumors [1-6]. The majority of the MitoChip detected mutations were somatic (dominant in tumor cells) and it has been suggested that these mutations may be used as markers for the early diagnosis of cancer [2]. However, many of these early stages MitoChip detected cancer mutations require accurate validation before put into routine clinical practice. Many recorded mtDNA mutations in cancer samples are not fully reliable. Employing a phylogenetic analysis of mtDNA tumor profiles taken from a specific example in the literature, we demonstrate the pitfalls of using MitoChip identified mitochondrial mutations for clinical diagnosis of premalignant cancers.

## Methods

The advent of MitoChip technology has allowed researchers to explore mtDNA for markers for primary cancers [1]. Mithani, Maitra, Sui, Zhou, and colleagues, for example, have extensively used the MitoChip platform for mutation detection in primary cancer samples and uncovered a potentially large number of somatic mutations [1-6]. To validate the recorded somatic mutations in cancer patients we compared their mtDNA sequence with the currently available complete mtDNA database [7-14]. Mitochondrial DNA (mtDNA) is exclusively maternally inherited, thus different mtDNA lineages cannot mix or recombine: the only way that the mtDNA sequence can change is by the sequential accumulation of mutations along radiating maternal lineages. Therefore, the mutations seen in tumors samples arise on a background of a fixed mitochondrial germline haplotype-defining mutation. With this general concept, we first traced the lineage associated mtDNA mutations observed in tumors and normal tissue samples by comparing the mutation motif with the published world mtDNA phylogenies [15]. Then, the mutations corresponding to haplotype lineages were paired with their normal tissue sample. If a tumor sample was distinguished from the corresponding normal tissue sample by the mutations that distinguish two different mtDNA lineages, then we could directly interpret that sample mix-up would produce the result, because the mutational processes involved in cancer could hardly produce, by chance, the mutations of evolutionary history.

## Results

**Figure 1:**
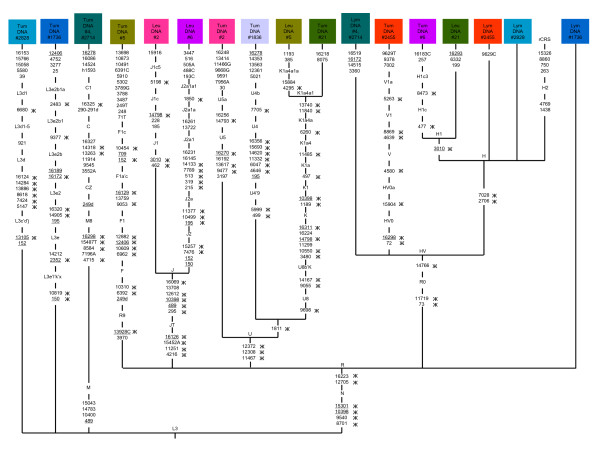
**The mtDNA phylogenetic tree constructed on tumor cases: The Head and Neck Squamous Cell Carcinoma-HNSCC patients #1736, #1836, #2455, #2714, and #2828 are from Zhou *et al. ***[3]**, and patients #2 and #4 are from Mithani *et al. ***[5]; **and the Adenoid Cystic Carcinoma-ACC patients #5, #6, and #21 are from Mithani *et al. ***[6]. All mutations are scored relative to the rCRS (revised Cambridge Reference Sequence) [20], prefix h indicates heteroplasmy and a suffix indicates a transversion, d-indicates deletion. Tum, Leu, and Lym-indicates Tumor, Leukocyte and Lymphocyte respectively. "Ж" signifies haplogroup specific variants which were found in the patients. Recurrent mutations are underlined. The names of haplogroups are given at the branching points or along single branches. Designation of haplogroups follows van Oven and Kayser [15].

Recently, one hundred forty two mitochondrial mutations were identified from 22 adenoid cystic carcinoma patients, among these, three patients (#5, #6, and #21, see Mithani *et al. *[6] supplementary table S1) accounted for 66% (94/142) of the total mutations [6]. More nonsynonymous amino-acid-changing mutations were found in the nicotinamide adenine dinucleotide dehydrogenase (NADH) complex suggesting that these changes play a role in tumor development. Surprisingly the tumor-related somatic mtDNA mutations belonged to an evolutionary pathway in the mtDNA phylogeny. The leukocyte mtDNA sequences of patient 5, 6, and 21 belong to haplogroups K1a4a1a, J2a1a1, and H1, whereas the respective tumor sequence mutation themselves belong to different haplogroups: F1c, H1c3, and K1a4a1 respectively (Figure 1). Clearly what we see here are not somatic mutations but different mtDNA lineages stemming from different individuals which were exchanged with each other and attributed to the patients in question.

Zhou *et al. *[3] sequenced the entire mitochondrial genome of 83 primary head and neck tumor samples (Head and Neck Squamous Cell Carcinoma-HNSCC), and identified 228 mutations by comparison with matched normal blood leukocyte DNA (see their supporting information table 2). Subsequently Mithani *et al. *[5] obtained the complete mtDNA sequence from salivary rinses of 13 HNSCC patients and found that salivary rinse mtDNA mutations were congruent with the sequenced leukocyte DNA (see their supplementary table 1), suggesting that salivary rinses of patients may also be useful in tumor risk assessment. Both the studies listed a large number of homoplasmic somatic changes, in particular sample numbers #2, #4, (Mithani *et al. *[5]), #1736, #1836, #2455, #2714, and #2828 (Zhou *et al. *[3]). Surprisingly, the phylogenetic analysis indicates that those listed tumor specific somatic mutations are known to follow the pathways of the African, Western Eurasian and East Asian mtDNA phylogeny. The samples #1736 and #2828 tumor sequence signify African lineages L3e2b1a and L3d1, whereas the leukocyte sequences follow the pathways of western Eurasian haplogroups R and H (Figure 1). Similarly, other tumor samples #2, #4, #1836, #2455, and #2714 sequences variants were found in the western Eurasian and East Asian haplogroup V1a, U4b, U5a, and C1; in contrast, leukocyte sequence information points to the haplogroups H, HV, J1c5, R (Figure 1). It seems in these cases, large numbers of homoplasmic mtDNA alterations found in HNSCC patients are best attributed to sample mix-up.

**Figure 2:**
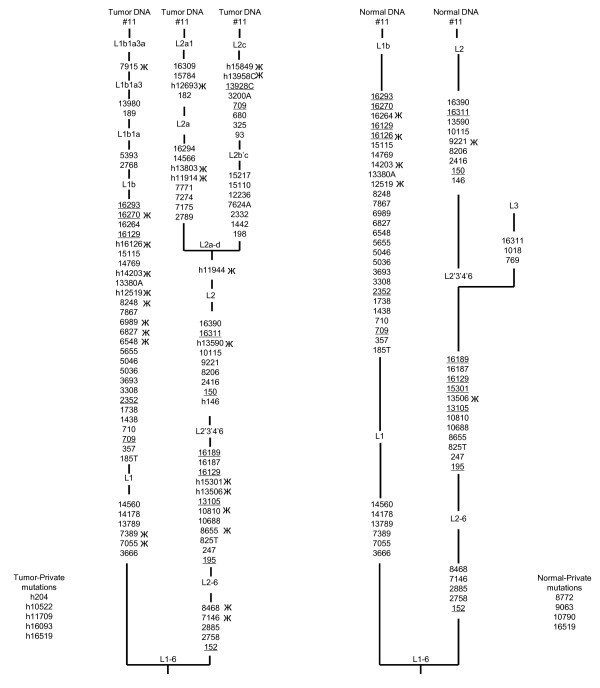
**The phylogenetic reconstruction of the sessile serrated adenoma (SSA) case 11 mtDNA sequence from Sui *et al. ***[2]. For additional information, see the fig. 1 legend.

Another paper by Sui *et al. *[2] aimed to demonstrate the existence of somatic mtDNA alterations in preneoplastic lesions of the gastrointestinal tract. In particular, they detected a high number (N = 35) of somatic mutations from a single sessile serrated adenoma patient (see their additional file 1, case 11), and add that their evidence supports the hypothesis that mtDNA alterations play a role in gastrointestinal neoplasia. However, we observe that many of the mtDNA alterations in tumor tissue are congruent with the African mtDNA haplogroups L1b1a3a (C6548T, T6827C, A6989G, A7055G, T7389C, C7915T, A8248G, C16270T and heteroplasmic mutations 12519, 12693, 14203, 16126) and L2a (A7146G, C8468T, C8655T, T10810C, and heteroplasmic mutations 11914, 11944, 13506, 13590, 13803, 15301), respectively. In addition, the heteroplasmic variants 13958 and 15849 are also found in the L2c haplogroup. Similarly, the mtDNA sequence of normal tissue showed mixed patterns. For example the four mutations - T12519C, A14203G, T16126C, C16264T have been found in haplogroup L1b lineages, and two other variants A9221G and C13506T have been recorded in haplogroup L2 lineages. Here, we see that sample mix-up generated the heteroplasmic and somatic mutations in case 11 (Figure 2). Furthermore, the authors observed one cytochrome oxidase subunit I polymorphism, - A7146G, in 3 adenomas (case 9, 10, and 11), and suggest a pathogenic role. Actually the heteroplasmic polymorphisms found in the case 9 and 10 result from mixing-up the samples of two lineages- L1b and L2, while the variant A7146G is belongs to the haplogroup L2. This effectively rules out a pathogenic role for A7146G polymorphism in adenomas.

Bandelt *et al. *[16] demonstrate another case of sample mix-up in Maitra *et al. *[1] data. The sample JHU-MITO # 12 tumor sequence belongs to haplogroup L3e3b, whereas lymphocyte sequence variants point to the haplogroup HV0a (refer Bandelt *et al. *[16] Figure 1) which clearly shows that two mtDNA lineages were attributed to the same patient. In addition, there are numerous cases from cancer research where the sample mix-up could have lead to the false detection of a high percentage of tumor-specific somatic mtDNA mutations, as Salas and colleagues have pointed out [16-18].

## Discussion

The use of mitochondrial DNA mutation and/or polymorphisms patterns to serve as markers is a rapidly expanding discipline in cancer clinical research [19]. With the advent of high-throughput techniques for the detection of mitochondrial DNA mutations such as MitoChip, increasingly researchers have been able to sequence entire mitochondrial genomes to promote an understanding of the mtDNA variants of tumor patients [1]. With MitoChip technology, a large number of somatic mutations in the mitochondrial genome have recently been reported in head and neck, adenoid cystic carcinoma, sessile serrated adenoma, and lung primary tumors [1-6]. The majority of the somatic mutations were reported from the nicotinamide adenine dinucleotide dehydrogenase (NADH) complex and many were nonsynonymous amino acid-changing mutations. The investigators of these studies then suggest as a mechanism that the high incidence of amino acid changing mutations in the NADH complex causes increased reactive oxygen species (ROS) production and subsequently increases mtDNA mutation and dysfunction, which creates conditions which favor tumors cell growth [2,3,6]. In fact from our phylogenetic analysis, we can see that those predicted mtDNA mutations in the tumor cases were actually the result of sample mix-up or contamination. It is an unfortunate fact that many clinical geneticists are not aware of existing mtDNA phylogenetic tools and their power to elucidate medical data. We emphasize that careful assessment of newly identified mtDNA sequence variants must be undertaken in clinical studies before the start of large scale tumor diagnostic mtDNA genotyping projects. While researchers put much effort in *a priori *quality control and it is also important to put equal effort into *a posteriori *control by performing phylogenetic analysis. In clinical oncogenetics studies-this could help establish which mtDNA variants are truly associated with tumors.

## Conclusions

Identifying mtDNA markers related to cancers could be very helpful, but data should be collected, analyzed, and interpreted with special care. Our analysis demonstrates that a significant proportion of recorded somatic mtDNA alterations in tumor patients are attributable to sample mix-up.

## Competing interests

The authors declare that they have no competing interests.

## Authors' contributions

MGP and YPZ designed the study, analyzed the data, and wrote the paper. All authors read and approved the manuscript.

## Pre-publication history

The pre-publication history for this paper can be accessed here:

http://www.biomedcentral.com/1471-2407/10/597/prepub

## References

[B1] Maitra A Cohen Y Gillespie SE Mambo E Fukushima N Hoque MO Shah N Goggins M Califano J Sidransky D Chakravarti A The Human MitoChip: a high-throughput sequencing microarray for mitochondrial mutation detection Genome Res 2004 14 812 9 10.1101/gr.2228504 15123581 PMC479107

[B2] Sui G Zhou S Wang J Canto M Lee EE Eshleman JR Montgomery EA Sidransky D Califano JA Maitra A Mitochondrial DNA mutations in preneoplastic lesions of the gastrointestinal tract: a biomarker for the early detection of cancer Mol Cancer 2006 5 73 10.1186/1476-4598-5-73 17166268 PMC1764424

[B3] Zhou S Kachhap S Sun W Wu G Chuang A Poeta L Grumbine L Mithani SK Chatterjee A Koch W Westra WH Maitra A Glazer C Carducci M Sidransky D McFate T Verma A Califano JA Frequency and phenotypic implications of mitochondrial DNA mutations in human squamous cell cancers of the head and neck Proc Natl Acad Sci USA 2007 104 7540 5 10.1073/pnas.0610818104 17456604 PMC1863503

[B4] Mithani SK Taube JM Zhou S Smith IM Koch WM Westra WH Califano JA Mitochondrial mutations are a late event in the progression of head and neck squamous cell cancer Clin Cancer Res 2007 13 4331 5 10.1158/1078-0432.CCR-06-2613 17671113

[B5] Mithani SK Smith IM Zhou S Gray A Koch WM Maitra A Califano JA Mitochondrial resequencing arrays detect tumor-specific mutations in salivary rinses of patients with head and neck cancer Clin Cancer Res 2007 13 7335 40 10.1158/1078-0432.CCR-07-0220 18094415

[B6] Mithani SK Shao C Tan M Smith IM Califano JA El-Naggar AK Ha PK Mitochondrial mutations in adenoid cystic carcinoma of the salivary glands PLoS One 2009 4 e8493 10.1371/journal.pone.0008493 20041111 PMC2795173

[B7] Herrnstadt C Elson JL Fahy E Preston G Turnbull DM Anderson C Ghosh SS Olefsky JM Beal MF Davis RE Howell N Reduced-median-network analysis of complete mitochondrial DNA coding-region sequences for the major African, Asian, and European haplogroups Am J Hum Genet 2002 70 1152 71 Erratum in: *Am J Hum Genet *2002, **71: **448-9 10.1086/339933 11938495 PMC447592

[B8] Palanichamy MG Sun C Agrawal S Bandelt HJ Kong QP Khan F Wang CY Chaudhuri TK Palla V Zhang YP Phylogeny of mitochondrial DNA macrohaplogroup N in India, based on complete sequencing: implications for the peopling of South Asia Am J Hum Genet 2004 75 966 78 10.1086/425871 15467980 PMC1182158

[B9] Sun C Kong QP Palanichamy MG Agrawal S Bandelt HJ Yao YG Khan F Zhu CL Chaudhuri TK Zhang YP The dazzling array of basal branches in the mtDNA macrohaplogroup M from India as inferred from complete genomes Mol Biol Evol 2006 23 683 90 10.1093/molbev/msj078 16361303

[B10] Kong QP Bandelt HJ Sun C Yao YG Salas A Achilli A Wang CY Zhong L Zhu CL Wu SF Torroni A Zhang YP Updating the East Asian mtDNA phylogeny: a prerequisite for the identification of pathogenic mutations Hum Mol Genet 2006 15 2076 86 10.1093/hmg/ddl130 16714301

[B11] Torroni A Achilli A Macaulay V Richards M Bandelt HJ Harvesting the fruit of the human mtDNA tree Trends Genet 2006 22 339 45 10.1016/j.tig.2006.04.001 16678300

[B12] Kivisild T Shen P Wall DP Do B Sung R Davis K Passarino G Underhill PA Scharfe C Torroni A Scozzari R Modiano D Coppa A de Knijff P Feldman M Cavalli-Sforza LL Oefner PJ The role of selection in the evolution of human mitochondrial genomes Genetics 2006 172 373 87 10.1534/genetics.105.043901 16172508 PMC1456165

[B13] Roostalu U Kutuev I Loogväli EL Metspalu E Tambets K Reidla M Khusnutdinova EK Usanga E Kivisild T Villems R Origin and expansion of haplogroup H, the dominant human mitochondrial DNA lineage in West Eurasia: the Near Eastern and Caucasian perspective Mol Biol Evol 2007 24 436 48 10.1093/molbev/msl173 17099056

[B14] Behar DM Villems R Soodyall H Blue-Smith J Pereira L Metspalu E Scozzari R Makkan H Tzur S Comas D Bertranpetit J Quintana-Murci L Tyler-Smith C Wells RS Rosset S Genographic Consortium The dawn of human matrilineal diversity Am J Hum Genet 2008 82 1130 40 10.1016/j.ajhg.2008.04.002 18439549 PMC2427203

[B15] van Oven M Kayser M Updated comprehensive phylogenetic tree of global human mitochondrial DNA variation Hum Mutat 2009 30 E386 94 10.1002/humu.20921 18853457

[B16] Bandelt HJ Kong QP Parson W Salas A More evidence for non-maternal inheritance of mitochondrial DNA? J Med Genet 2005 42 957 60 10.1136/jmg.2005.033589 15923271 PMC1735965

[B17] Salas A Yao YG Macaulay V Vega A Carracedo A Bandelt HJ A critical reassessment of the role of mitochondria in tumorigenesis PLoS Med 2005 2 e296 10.1371/journal.pmed.0020296 16187796 PMC1240051

[B18] Bandelt HJ Salas A Contamination and sample mix-up can best explain some patterns of mtDNA instabilities in buccal cells and oral squamous cell carcinoma BMC Cancer 2009 9 113 10.1186/1471-2407-9-113 19371404 PMC2678148

[B19] Barker PE Biomarker validation for aging: lessons from mtDNA heteroplasmy analyses in early cancer detection Biomarker Insights 2009 4 165 79 20029650 10.4137/bmi.s2253 PMC2796862

[B20] Andrews RM Kubacka I Chinnery PF Lightowlers RN Turnbull DM Howell N Reanalysis and revision of the Cambridge reference sequence for human mitochondrial DNA Nat Genet 1999 23 147 10.1038/13779 10508508

